# Study the Longitudinal *in vivo* and Cross-Sectional *ex vivo* Brain Volume Difference for Disease Progression and Treatment Effect on Mouse Model of Tauopathy Using Automated MRI Structural Parcellation

**DOI:** 10.3389/fnins.2019.00011

**Published:** 2019-01-24

**Authors:** Da Ma, Holly E. Holmes, Manuel J. Cardoso, Marc Modat, Ian F. Harrison, Nick M. Powell, James M. O’Callaghan, Ozama Ismail, Ross A. Johnson, Michael J. O’Neill, Emily C. Collins, Mirza F. Beg, Karteek Popuri, Mark F. Lythgoe, Sebastien Ourselin

**Affiliations:** ^1^Translational Imaging Group, Centre for Medical Image Computing, University College London, London, United Kingdom; ^2^Centre for Advanced Biomedical Imaging, University College London, London, United Kingdom; ^3^School of Engineering Science, Simon Fraser University, Burnaby, BC, Canada; ^4^School of Biomedical Engineering and Imaging Sciences, King’s College London, London, United Kingdom; ^5^Tailored Therapeutics, Eli Lilly and Company, Lilly Corporate Center, Indianapolis, IN, United States; ^6^Eli Lilly & Co. Ltd., Erl Wood Manor, Windlesham, United Kingdom

**Keywords:** *in vivo*, *ex vivo*, structural parcellation, longitudinal, disease progression, treatment effect, volumetric, atlas-based segmentation

## Abstract

Brain volume measurements extracted from structural MRI data sets are a widely accepted neuroimaging biomarker to study mouse models of neurodegeneration. Whether to acquire and analyze data *in vivo* or *ex vivo* is a crucial decision during the phase of experimental designs, as well as data analysis. In this work, we extracted the brain structures for both longitudinal *in vivo* and single-time-point *ex vivo* MRI acquired from the same animals using accurate automatic multi-atlas structural parcellation, and compared the corresponding statistical and classification analysis. We found that most gray matter structures volumes decrease from *in vivo* to *ex vivo*, while most white matter structures volume increase. The level of structural volume change also varies between different genetic strains and treatment. In addition, we showed superior statistical and classification power of *ex vivo* data compared to the *in vivo* data, even after resampled to the same level of resolution. We further demonstrated that the classification power of the *in vivo* data can be improved by incorporating longitudinal information, which is not possible for *ex vivo* data. In conclusion, this paper demonstrates the tissue-specific changes, as well as the difference in statistical and classification power, between the volumetric analysis based on the *in vivo* and *ex vivo* structural MRI data. Our results emphasize the importance of longitudinal analysis for *in vivo* data analysis.

## Introduction

In neuroimaging studies, quantitative analysis of neuroanatomy, such as volumetric analysis of brain structures extracted from magnetic resonance imaging (MRI) data sets, plays a crucial role in the diagnosis of diseases at the early stages of pathology before the onset of clinical symptoms (McEvoy and Brewer, 2010). This has been facilitated by automated analysis techniques such as atlas-based parcellation, which enable large data sets to be analyzed in a time efficient and unbiased manner. The application of MRI to study mouse models is increasingly being utilized to understand disease mechanisms as well as potential treatment effects, and a number of mouse brain MRI atlases are currently in existence to facilitate structural analysis of these models (Ma et al., 2005, 2008, 2014; Bai et al., 2012). However, whether to acquire data *in vivo* or *ex vivo* is always a debatable question during experimental design. For brains scanned *ex vivo*, there are no motion artifacts, and the prolonged scanning time enables (1) increased image resolution (leading to less partial volume effects), (2) improved signal to noise ratio, and (3) enhanced tissue contrast (Montie et al., 2010; Lerch et al., 2012; Holmes et al., 2017). The quality of images acquired *ex vivo* can be further enhanced using high concentrations of contrast-enhancement agents such as Gadolinium (Cleary et al., 2011). The enhancement of image quality in the *ex vivo* data can increase the statistical power to detect subtle volume changes when performing cross-sectional comparison between normal and disease groups (Lerch et al., 2012). However, samples prepared for *ex vivo* imaging suffer from morphological disruption to the tissues during processes such as fixation and perfusion (Lavenex et al., 2009). On the other hand, most of the intrinsic physiological and pathological characteristics of the animal’s tissues can be preserved if they are imaged *in vivo* (Schulz et al., 2011). Furthermore, with *in vivo* imaging, it is possible to trace the morphological changes of each individual animal longitudinally. This is especially important for monitoring disease progression (Zhang et al., 2010), as well as potential treatment effects over time using transgenic mouse models (Santacruz et al., 2005; Holmes et al., 2016). The trade-offs between longitudinal *in vivo* and cross-sectional *ex vivo* imaging data are an important factor to be consider during experimental design.

Our current understanding of volume changes from *in vivo* to *ex vivo* is inconclusive. Studies show inconsistent results on both clinical (Schulz et al., 2011; Kotrotsou et al., 2014) and preclinical imaging data (Ma et al., 2005, 2008; Zhang et al., 2010; Oguz et al., 2013). Lerch et al. (2012) measured the theoretical statistical power to compare *in vivo* and *ex vivo* imaging. Meanwhile, Holmes et al. (2017) investigated the effect size and sample size required for data analysis using tensor-based morphometry (TBM). In this study, we aim to further study and compare the volumetric analysis of individual structures using either longitudinal *in vivo* data or single-time-point *ex vivo* data acquired on the same animals.

Accurate structural parcellation is crucial for volumetric analysis. Conventional methods used to obtain volumetric information for regions-of-interest (ROIs) routinely implement manual delineation methods, which are both time-consuming and prone to human error (Ma et al., 2005; Richards et al., 2011). Comparatively, automatic structural parcellation has been continually improved and increasingly adopted to overcome the disadvantages of manual methods (Calmon and Roberts, 2000; Sharief et al., 2008; Almhdie-Imjabber et al., 2010). Recently, multi-atlas based techniques have been shown to provide highly accurate structural volumes in both clinical and preclinical studies (Rohlfing et al., 2004; Warfield et al., 2004; Aljabar et al., 2007; Cardoso et al., 2013; Ma et al., 2018).

In this study, we compared structural volumetric information extracted from both *in vivo* and *ex vivo* mouse brain data sets using a fully automated multi-atlas structural parcellation framework (Ma et al., 2014). We sought to explore how changes in volumes between *in vivo* and *ex vivo* in the mouse brain are distributed across different brain tissues and structures; whether the difference varies across different strains and treatment; and whether those variations within structures affect the statistical and classification power when comparing volumetric differences with expected pathology changes of brain atrophy with and without drug treatment. We also investigated whether including longitudinal information can improve the analysis of group differences.

## Materials and Methods

### Experimental Data

We used the rTg4510 transgenic mouse strain, which faithfully recapitulates several key features of clinical Alzheimer’s disease (AD) and frontal temporal dementia (FTD) including progressive atrophy of the forebrain regions and the accumulation of neurofibrillary tangles of tau (NFTs) (Santacruz et al., 2005). The NFT overexpression level and accompanying volumetric brain changes in the rTg4510 mouse can be attenuated using doxycycline, (Holmes et al., 2016); thus, this mouse model offers a unique paradigm to test the sensitivity of the analysis toward the level of structural changes.

17 rTg4510 and 8 litter-matched wild-type controls were bred on a mixed FVB/NCrl + 129S6/SvEvTa background for Eli Lilly and Company by Taconic (Germantown, MD, United States) and received on site 2 weeks before the initiation of the study. Only female mice were included to control the effect of sex differences. The rTg4510 mouse model exhibit early and fast progressing tau pathology (Santacruz et al., 2005), with mature NFTs observable between 3 and 5.5 months (Yue et al., 2011) and rapid progressing neuronal loss in the CA1 region of hippocampus by 5.5 months of age (Santacruz et al., 2005; Spires et al., 2006). Therefore, out of the 17 rTg4510, 10 received no intervention (untreated group), and the remaining 7 were administered with doxycycline from 3.5 months of age to coincide with early NFT formation, and enable potential treatment effects to be studied in both the *in vivo* and *ex vivo* data sets.

Longitudinal *in vivo* scans were performed at age of 4.5 months, 5.5 months, and 7.5 months to capture disease progression and doxycycline treatment in the corresponding groups. T2-weighted images were acquired using a 3D fast spin-echo sequence with a 72 mm birdcage radiofrequency (RF) coil. The animals were sacrificed immediately after the 7.5 months *in vivo* scan, to enable a direct comparison of structural brain changes from the *in vivo* and *ex vivo* data sets. An active staining technique was used to enhance the contrast for *ex vivo* imaging, by perfuse-fixing the animals using buffered formalin saline doped with 8 mM Magnevist, and soaking the decapitated brains in-skull at 4°C in this solution for 9 weeks prior to imaging (Cleary et al., 2011). A 35 mm birdcage RF coil was used for *ex vivo* imaging. The *in vivo* and *ex vivo* images were scanned using different RF coils and imaging gradient sets. The gradient scaling errors and non-linearity was calibrated to eliminate scaling effects (O’Callaghan et al., 2014). The detailed *in vivo* and *ex vivo* scanning protocols can be found in Holmes et al. (2017). The resolution of the *in vivo* and *ex vivo* images was 150 μm isotropic and 40 μm isotropic, respectively.

### Automatic Structural Parcellation

Brain structures were extracted using the multi-atlas segmentation propagation framework, which has been validated on both *in vivo* and *ex vivo* mouse brain MRI data and demonstrated accurate segmentation results (Ma et al., 2014; Powell et al., 2016). We adopted a publicly available MRM NeAt atlas database created by Ma et al. (2008) which includes 35 manual labeled anatomical structures for 10 *in vivo* and 10 *ex vivo* images (Ma et al., 2005) with structure labels created using the same manual segmentation protocol. The left/right hemispheres were automatically separated as described in Ma et al. (2014) to make them more biologically plausible.

In the preprocessing step, the test images were first reoriented to the same orientation of the atlas (PLS), and then corrected for intensity inhomogeneities using the N4 algorithm (Tustison et al., 2010). The images from the atlas were then registered to the pre-processed test images, first globally with a symmetric block-matching affine approach (Ourselin et al., 2000; Modat et al., 2014), followed by a local non-rigid registration step with asymmetric scheme based on a cubic B-Spline parametrization of a stationary velocity field and similarity measurements based on normalized mutual information (Rueckert et al., 1999; Modat et al., 2014). A deformation map between each atlas image and test image pair was generated from the image registration, which was then applied to transform the corresponding manually segmented brain structural labels of the atlas image to the test image space. The normalized mutual information ensures that the image similarity measurement is insensitive to the intensity profile difference between the registered image pairs (Rueckert et al., 1999). Gradient descent optimization was implemented to maximizing the image similarity, and the global (affine) to local (non-rigid) registration framework help to prevent the optimization scheme from been caught in the local minimum (Crum et al., 2004). The registered structural labels were ranked and fused using local normalized cross-correlation similarity measurements to obtain the best consensus structure label (Cardoso et al., 2013).

Careful quality assurance (QA) was performed on each automatically generated brain mask, which is the summation of all the parcellated structural labels. Manual corrections of the brain mask were applied on regions where voxels of external CSF were sometimes misclassified as brain tissue at the edge of the brain mask. The misclassified voxels happened mostly in the data from the untreated transgenic groups (for both *in vivo* and *ex vivo*), when the shrinkage of the brain tissues induced excessive amounts of external CSF to accumulate in the subarachnoid space. This phenomenon mostly appeared in the posterior part of the brain. Post-QA, the volume of each brain structure was extracted from the parcellation result with corrected brain mask.

The resolution of the *ex vivo* data is higher than the *in vivo* data because of the longer image acquisition time, the T1-shortening effects of the contrast agent, and the use of a smaller imaging gradient set; in order to eliminate effects simply due to the difference in image resolution, we also down-sampled the *ex vivo* images from the original resolution (40 μm) to the same resolution of the *in vivo* image (150 μm) with spline interpolation, and applied the same multi-atlas structural parcellation pipeline using the same atlas.

### Gray-Matter/White-Matter Contrast-to-Noise Analysis

We compared the gray-matter/white-matter (GM/WM) contrast-to-noise ratio (CNR) between the *in vivo* and *ex vivo* images, for each of the treated and untreated rTg4510 groups, and the wild-type controls, using the following formula: CNR = (Siginal_GM_ − Signal_WM_)/Noise. We grouped the labels for all the GM structures as well as for all the WM structures and measured the mean intensity across the entire GM and WM regions accordingly as their signal intensities. To measure the background noise, we first affinely registered images of all the subjects to a common groupwise space by randomly selecting one subject as the reference. We took the average of all the affinely registered images and manually defined a region of interest (ROI) in the image background which doesn’t contain any tissue signals and is ghost-free. We then propagated the ROI back to all the subjects by taking the inverse transform of the affine matrix generated from the groupwise registration. The noise for each image was then measured as the standard deviation of the propagated background ROI. Manual QA was performed to ensure the propagated ROI was located in the background for all subjects. The background noise for each image was then defined as the standard deviation within the background ROI. We compared the CNR with an unpaired one-tail Student *t*-test. Multiple comparisons were corrected with a false discovery rate (FDR) of 0.05 (Chumbley and Friston, 2009; Storey, 2010).

### Structure Volume Comparison of Between *in vivo* and *ex vivo* Measurement

Subsequently, we used the Bland–Altman analysis to investigate the proportional differences in structural volumes measured from *in vivo* and *ex vivo* data at the same time-point (7.5 months) in order to explore the local variation of volume changes across structures using the automatically parcellated structural labels. To control for partial volume effects due to the resolution difference, we compared the *in vivo* structure volume to the down-sampled *ex vivo* volume to ensure same resolution (150 μm).

The Bland–Altman plot is often the method of choice in medical research for measuring the agreement or difference between two measurements (Altman and Bland, 1983; Martin Bland and Altman, 1986; Myles and Cui, 2007). It is recommended by Pollock et al. (1992) that, when the variability of the measurement differences is related to the magnitude of the measurements, one should plot the proportional difference to the magnitude of the measurements on the *y*-axis of the Bland–Altman plot instead of the absolute difference. In this study, the difference in the measured volume should be represented as the proportional of the underlying structural size. Therefore, we plotted the percentage volume difference (PVD) between structural volumes as a proportion of the mean structure size (Eq. 1). For each structure:

(1) $$\mathrm{PVD}=\frac{V_{\mathrm{ex}}-V_{\mathrm{in}}}{(V_{\mathrm{ex}}+V_{\mathrm{in}})/2}\times 100\%$$

where *V*_in_ and *V*_ex_ are the individual structure volumes extracted from *in vivo* and *ex vivo* brains, respectively.

We compared the *in vivo* and *ex vivo* measurements for each structure across all subjects within all groups through paired *t*-tests for all the parcellated structures to investigate whether the observed volume differences were statistically significant. Multiple comparisons were corrected with FDR = 0.05. We also compared the mean *in/ex vivo* structural volume differences among the three different groups using an analysis of variance (ANOVA) followed by Bonferroni *post hoc* test to compensate for multiple tests for each structure. Multiple comparisons across different structures were further controlled with FDR set to 0.05.

### Group Difference Analysis

Volumetric analysis is often used as a surrogate imaging biomarker to distinguish subjects from different groups. In the next step, we assessed and compared the statistical analysis results measuring the group difference using the parcellated structures from the *in vivo* data and that from the *ex vivo* data. We included only the rTg4510 transgenic animals in this step, in order to control for effects due to genetic differences. We compared the brain structures between the untreated and the doxycycline-treated rTg4510 groups. The structure volume is normalized to the total brain volume (TBV) by modeling the volume as a linear combination of the TBV and the residual term (Eq. 2), then fitting the linear model to the data from the untreated group (regarded as the reference group) and taking the standardized residual (w-score) as the measured feature (Eq. 3). The residual-based structure normalization method has been proved to be more effective at removing the confounding effect of TBV compared to the proportional method which achieves the normalization through simply dividing the structure volume by the TBV (Sanfilipo et al., 2004). The w-score is the recommended method for evaluating the structure changes such as atrophy (O’brien and Dyck, 1995; La Joie et al., 2012; Collij et al., 2016; Ma et al., 2018). It is equivalent to the z-score of the residual showing the difference of each volume measurements when comparing to the reference group mean. Therefore, the difference in w-score represent the difference in pathological severity, effectively reflecting the treatment effect of doxycycline. We performed unpaired two-tailed *t*-tests on the normalized volumes of all the parcellated structures between the untreated and doxycycline-treated rTg4510 groups, for both the *in vivo* and *ex vivo* data. All tests were corrected for multiple comparisons with a FDR of 0.05. Multiple comparisons were corrected with a FDR of 0.05.

(2) $$V_{i}=\beta_{0}+\beta_{1}T_{i}+\varepsilon_{i}$$

where *V_i_* is the raw structure volume for subject *i, T_i_* is the corresponding TBV, ε_*i*_ is the residual term. The normalized volume ${\hat{V}}_{i}$ (w-score *w_i_*) is calculated as:

(3) $${\hat{V}}_{i}=w_{i}=\frac{\varepsilon_{i}-\mu_{\varepsilon UT}}{\sigma_{\varepsilon UT}}$$

where μ_ε_CN__ and σ_ε_UT__ are the mean and standard deviation of the residual for the untreated (reference) group.

It has been shown that incorporating longitudinal data can theoretically improve the classification power of the data (Lerch et al., 2012; Kim and Kong, 2016). Therefore, we also estimated the longitudinal structure volume change rate to evaluate whether the longitudinal information obtained from the *in vivo* scans provide complementary information over the single-timepoint data sets. The longitudinal structure volume change rate is estimated by fitting a linear model to the longitudinal volume data from the three time-points (3.5, 4.5, and 7.5 months), as shown in Eq. (4).

(4) $$V_{j}^{t_{i}}=V_{j}^{t_{0}}+R_{j}\times (t_{i}-t_{0})+\varepsilon$$

Where $V_{j}^{t_{i}}$ is the measured volume of structure *j* at time *t_i_*, the slope parameter *R_j_* represent the volume change rate of structure *j*, and ε is the error term. Unpaired *t*-tests were performed to compare the structural change rate between the treated and untreated group.

### Evaluation of the Classification Power

In the last step, we compared the classification power between the *in vivo* and *ex vivo* volume measurements. Again, we included the untreated and doxycycline-treated groups of mice, all from the same genetic background (rTg4510). We used a support vector machine (SVM) with a linear kernel as the classifier to classify the treated and untreated groups. All parcellated structures were regarded as features for classification, and all features were scaled to the mean ± 1 SD. Due to the small sample size, threefold cross-validation was conducted. In each fold, we evaluated the ability of the model to correctly classify the mice in the test set based on the pre-classified training set. Feature dimensions were reduced using principal component analysis (PCA), with the number of principal components fed to the classifier chosen to represent 95% of the total variance of the training set. We evaluated the classification performance using the mean area under the curve (AUC) of the receiver operating characteristic (ROC), with a larger mean AUC representing better classification power.

For *in vivo* data, our evaluations include: (a) only the third timepoint data (7.5 months); (b) the longitudinal data (in the form of absolute structural change rate); and (c) the combined feature including both the third time-point normalized structure volume as well as the longitudinal absolute structural volume change rate. For the *ex vivo* data, we evaluated the classification power for both the original and the down-sampled data.

In order to study the effect of the sample size toward the power to classify the treated and untreated group of the SVM classifier for both *in vivo* and *ex vivo* data, we plotted the learning curve which shows the changes of classification accuracy of both training set and cross-validation test set with different sample size (Figueroa et al., 2012; Beleites et al., 2013). We performed the sample size analysis for all five sets of data: (a) the third timepoint *in vivo* data (7.5 months); (b) the longitudinal *in vivo* data; (c) the combined feature including both the single timepoint and longitudinal *in vivo* data; (d) the raw *ex vivo* data; and (e) the down-sampled *ex vivo* data.

### Evaluation Longitudinal Individual Variation

Furthermore, we also investigated the selection of timepoint that reflects the longitudinal trend of pathology manifest and the treatment effect based on the volumetric *in vivo* data. We focused our analysis specifically on three structures – hippocampus, cortex, and ventricle – given that cortical and hippocampal atrophy, as well as ventricle expansion, are widely accepted biomarkers for AD-related pathology (Thompson et al., 2001, 2004; Holmes et al., 2016; Rathore et al., 2017). The volume differences among three groups at each timepoints were compared using ANOVA test followed with Bonferroni *post hoc* test to test statistical difference between each group pairs. Multiple comparisons were corrected with FDR = 0.05.

In addition, it is important to address the individual variation in biomedical experiment, especially for longitudinal analysis (Klingenberg, 1996; Roche et al., 2016). We used linear mixed-effect model (LME) (Roche et al., 2016; Lee et al., 2018) to evaluate the individual variation across the timepoints for all three groups (Eq. 5). The individual variance is modeled in three different ways:

(a) The longitudinal measurements for each individual subject are modeled as fixed-term (Eq. 5.1), without explicitly modeling of the individual variation;
(b) Individual volume variance was explicitly model by introducing a random-effect term on the intercept (Eq. 5.2);
(c) Individual variance on the longitudinal volume change was also modeled by including an additional random-effect term on the slope of time (Eq. 5.3).

(5) $$V_{i}=\beta_{0}+\beta_{1}\;(\mathrm{time})+\beta_{2}\;(\mathrm{group})+\beta_{3}\;(time\;\times \;group)+\beta_{4}\;(subject)+\varepsilon_{i}$$

(6) $$V_{i}=\beta_{0}+\beta_{1}\;(\mathrm{time})+\beta_{2}\;(\mathrm{group})+\beta_{3}\;(time\;\times \;group)+b_{1,i}+\varepsilon_{i}$$

(7) $$V_{i}=\beta_{0}+\beta_{1}\;(\mathrm{time})+\beta_{2}\;(\mathrm{group})+\beta_{3}\;(time\;\times \;group)+b_{1,i}+b_{2,i}\;(\mathrm{time})+\varepsilon_{i}$$

where *V_i_* is the structure volume for subject i, β_0_ represent the intercept term, β_1_ represent the fixed-effect of time (or animal age), β_2_ represent the fixed-effect of the three experimental groups, β_3_ represent the interaction of group with time, β_4_ in Eq. (5.1) represent the modeled fixed-effect of individual subject as a grouping term, b_1*i*_ in Eqs. (5.2) and (5.3) represent the modeled random-effect of individual variance on the intercept, b_2*i*_ in Eq. (5.3) represent the modeled random-effect of individual variance on the longitudinal scale, and ε_*i*_ is the residual error in the model.

We use restricted maximized likelihood (REML) to fit each model and use the Akaike information criterion (AIC) to determine and compare the model performance. To demonstrate the model improvement after considering the individual variation as the random effect, we also fit each model to the original data, and calculated the individual residual as the difference between the model-predicted volume and the true volume. We compared the relative residual, calculated as the ratio between the fitted residual and the actual measured volume among the three models, for the three selected structures in all three groups across different timepoints.

## Results

### Automatic Structural Parcellation

**FIGURE 1:**
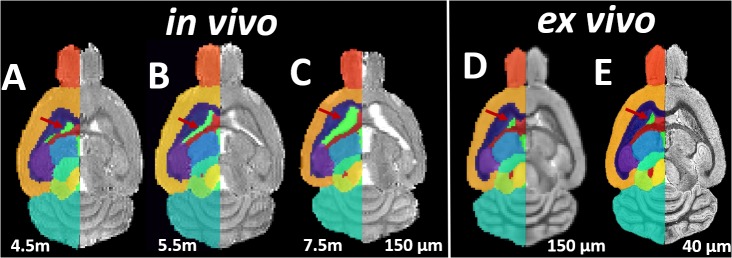
Representative axial slices of the Longitudinal *in vivo* and *ex vivo* images of the untreated transgenic mice, overlaid with the automatic parcellated structural labels. **(A–C)** *In vivo* images acquired at 4.5, 5.5, and 7.5 months. **(D)** The *ex vivo* image down-sampled to the same resolution of *in vivo* image (150 μm). **(E)** The *ex vivo* image with the original resolution (40 μm). Red Arrow: the longitudinal *in vivo* expansion and the *ex vivo* collapse of the ventricle is accurately delineated by parcellated labels.

Both the *in vivo* and *ex vivo* mouse brain images were segmented accurately into 35 anatomical structures as defined in the MRM NeAt mouse brain atlas, using the automated structural parcellation framework described in the “Materials and Methods” section. Figure 1 shows the representative images of the untreated transgenic mouse, overlaid with the corresponding automatic parcellated structures, including the longitudinal *in vivo* images (Figures 1A–C) as well as the *ex vivo* images with both the down-sampled (Figure 1D) and the original resolution (Figure 1E). Visual inspection revealed that the parcellated structures accurately align with the anatomy, showing morphological differences between the *in vivo* and *ex vivo* images. The longitudinal expansion of the ventricles (*in vivo*) and the collapse of the ventricles (*ex vivo*; as shown in the red arrows), can be readily visualized. Table 1 shows the comparison of GM/WM CNR between the *in vivo* and *ex vivo* images. The *ex vivo* images exhibit superior tissue CNR compared to the *in vivo* images for animals in all groups.

**Table 1:** Comparison of GM/WM tissue contrast-to-noise-ratio (CNR) between the *in vivo* and *ex vivo* images.

CNR	all	wildtype	treated transgenic	untreated transgenic
*In vivo*	1.07 ± 0.22	1.32 ± 0.12	0.93 ± 0.10	1.00 ± 0.17
*Ex vivo*	2.46 ± 0.15	2.40 ± 0.13	2.50 ± 0.13	2.46 ± 0.18
*p*-value	<0.001^∗^	<0.001^∗^	<0.001^∗^	<0.001^∗^


*The *ex vivo* images showed significant higher CNR compared to the *in vivo* images for animals in all groups. ^∗^Statistical significant was observed with *p*-value smaller than 0.001.*

### *In vivo* to *ex vivo* Volumetric Difference

**FIGURE 2:**
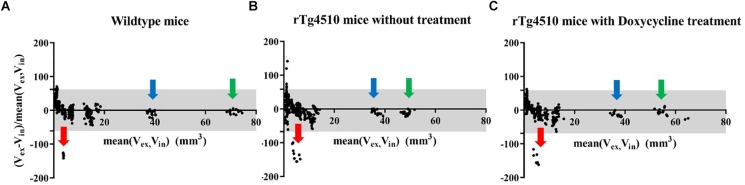
Bland–Altman plot showing the structure volume difference in the *in vivo* and *ex vivo* volume measurement (proportional to the mean volume of the two measurements). Gray area: 95% limit of agreement between *in vivo* and *ex vivo* measurements. **(A)** FVB/NCrl wild-type mice. **(B)** rTg4510 mice without treatment. **(C)** rTg4510 mice with doxycycline treatment. Arrows show examples of three distinctive structures, red arrow: ventricle; blue arrow: cerebellum; green arrow: neocortex.

Firstly, we compared the pair of *in vivo* and *ex vivo* structure volumes both acquired at 7.5 months. The *ex vivo* data were down-sampled to the same resolution as the *in vivo* data (150 um) to control the effect comes from the resolution difference. Figure 2 shows the results of the Bland–Altman analysis for the (Figure 2A) wild-type controls, (Figure 2B) the untreated rTg4510 group, and (Figure 2C) the doxycycline-treated rTg4510 group. The >100% relative volume shrinkage of the ventricles (Figure 2; red arrow) reflects the collapse of the ventricles from *in vivo* to *ex vivo*. The Bland–Altman plot shows variations in volume difference, indicating a non-linear non-uniform distribution of the volume shrinkage from *in vivo* to *ex vivo*.

**FIGURE 3:**
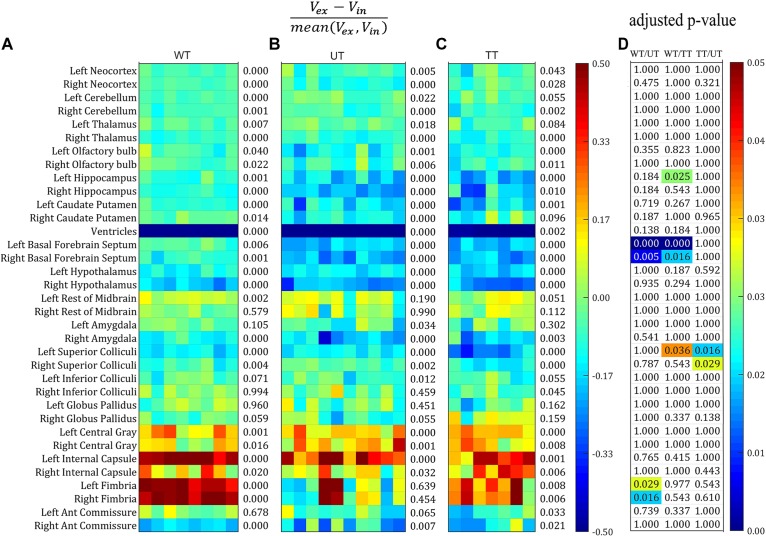
The percentage volume difference of each structure calculated from the Bland–Altman analysis for all the individual mouse across all three groups. Structures were listed from large to small top down. **(A–C)** The value were thresholded to within the range of [–0.5, 0.5]. The number at the right of each subplot represent the adjusted *p*-value of the paired *t*-test between the *in vivo* and *ex vivo* measurement (multiple comparisons were corrected with FDR = 0.05). **(A)** Wild-type group (WT). **(B)** Transgenic group without doxycycline treatment (untreated, UT). **(C)** Transgenic group with doxycycline treatment (treated, TT). **(D)** The adjusted *p*-value of the pairwise comparison among all three groups after ANOVA with Bonferroni *post hoc* test followed by multiple comparison corrections with FDR = 0.05. Only the significant *p*-values were shown with color (ranging from [0, 0.05]).

We then plotted the percentage volume change as calculated from Bland–Altman analysis of all structure for each individual mouse in all three groups: the wild-type group, the untreated rTg4510 group, and the doxycycline-treated rTg4510 group (Figures 3A–C). The structures are listed in descending order of size: the top 29 structures are gray matter structures (except for the ventricles); the bottom 6 structures are white matter: (internal capsule, fimbria, and anterior commissure). We also performed paired *t*-test between the volume measured both *in vivo* and *ex vivo* for each group. The number at the right of each subplot represent the adjusted *p*-value of the paired *t*-test between the *in vivo* and *ex vivo* measurement (multiple comparisons were corrected with FDR = 0.05). Significant level of *in/ex vivo* differences are observed in most structures for all three groups, and the ventricular collapses are apparent for all groups (shown as the dark blue band), reflecting widespread changes induced by the preparation of the tissues for *ex vivo* scanning (Figures 3A–C). The *ex vivo* volumes were significantly smaller for the majority of the gray matter structures (e.g., neocortex, cerebellum, thalamus, olfactory bulb, hippocampus, caudate putamen, basal forebrain septum, hypothalamus, amygdala and superior/inferior colliculi) except for the central gray (the smallest labeled gray matter structure), which exhibited a significantly larger *ex vivo* volume compared to *in vivo* volume. On the other hand, most of the white structures demonstrated significantly larger *ex vivo* volumes than *in vivo* volumes (i.e., internal capsule, and fimbria) except for the smallest white matter structure, the anterior commissure, which was significantly smaller *ex vivo*. For the structure labeled “rest of midbrain” where there is a mix of white and gray matter, the volume change is not significant.

Figure 3D shows the statistical results of the ANOVA analysis, comparing the mean *in/ex vivo* volume difference among three groups (with Bonferroni *post hoc* test followed by multiple comparison corrections with FDR = 0.05). The majority of volume differences were not significantly different between groups; however, significant differences were detected for: the hippocampus (left side only) when comparing the wild-type controls to the treated rTg4510 group; the basal forebrain septum when comparing wild-type group to the rTg4510 groups (both the treated and untreated); the superior colliculi when comparing both the treated and untreated rTg4510 groups, as well as the wild-type to untreated rTg4510 group (left side only); and the fimbria when comparing between wild-type to the treated transgenic group (all shown in Figure 3D).

### Group Difference Analysis

**FIGURE 4:**
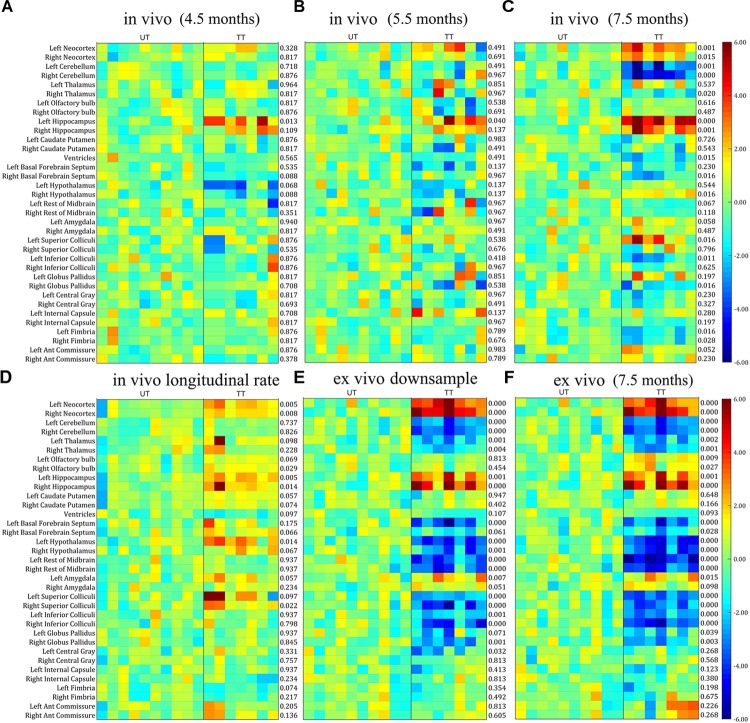
The w-score of the TBV normalized volume for each structure across subjects for both untreated (UT) and Doxycycline treated (TT) groups, with the untreated group as the reference group. The number at the right of each subplot represent the statistical results of unpaired two-tailed *t*-tests comparing the normalized structural volume of the treated and untreated group on both *in vivo* and *ex vivo* data. **(A–C)** *In vivo* data at different time-points (3.5, 5.5, and 7.5 months). **(D)** The longitudinal volume change calculated from the *in vivo* data at three time-points. **(E)** *Ex vivo* data down-sampled to the same resolution of the *in vivo* data (150 μm). **(F)** Original *ex vivo* data acquired at a resolution of 40 μm. All tests were corrected for multiple comparisons with a false discovery rate (FDR) of 0.05.

Next, we investigated whether the differences in *in vivo* and *ex vivo* volume measurements affected the statistical analysis when analyzing the treatment effect, by comparing the parcellated volumes of rTg4510 mice with and without doxycycline treatment. The structural volumes were normalized to TBV using the standardized residual (w-score), as described in the “Materials and Methods” section. Figure 4 shows the w-score of the volume for each structure across subjects for both rTg4510 groups (with untreated titled as UT, and treated group titled as TT), with the untreated group as the reference group. The w-score of each structure shows the difference between the normalized structure volume of the subject to the reference group mean, normalized by the reference group standard deviation. The number at the right of each subplot represents the adjusted *p*-value when comparing the untreated and treated group with two-tailed unpaired *t*-test. We performed the group difference analysis on both the single time-point data, as well as the longitudinal data, which are described in detail below.

#### Single Time-Point Analysis

In order to make a direct comparison between structural changes identified *in vivo* versus *ex vivo*, we first compared the statistical analysis between *in vivo* and *ex vivo* data acquired at the same 7.5 months’ time-point. The *in vivo* results (Figure 4C) revealed a significant reduction in ventricle size after doxycycline treatment; however, this finding was not detected in the *ex vivo* data (Figure 4F) due to the ventricular collapse during the preparation of the post-mortem tissues. For the white matter (Figure 4; bottom six rows of each subplot), no significant volume differences were detected in any of the *ex vivo* white matter regions (Figure 4F), but a significant volume decrease was detected in the fimbria in the *in vivo* data (Figure 4C).

Within the gray matter, the *in vivo* data (Figure 4C) showed significant volume increases in the neocortex and hippocampus, right hypothalamus, left superior colliculi, and significant volume decreases in the right thalamus, right basal forebrain septum, left inferior colliculi and right globus pallidus. The statistical analysis of the *ex vivo* volumetric data acquired at the same time (Figure 4F) showed a similar pattern of group differences. However, the *ex vivo* volume analysis revealed additional significant volume decreases in the left thalamus, the olfactory bulb, the left basal forebrain septum, hypothalamus, superior/inferior colliculi, central gray (for both the raw and down-sampled data), and a significant volume increase in the left amygdala, which was not shown in the *in vivo* volumetric data. Interestingly for the hypothalamus, the *in vivo* results revealed an increase in volume within the hypothalamus associated with doxycycline treatment, while the *ex vivo* data showed a volume decrease. These discrepancies highlight the potential confounding effects of post-mortem tissue processing on *ex vivo* structural volumes. In addition, both the *in vivo* (Figure 4C) and *ex vivo* data showed significant cerebellar volume shrinkage after the doxycycline treatment (the third and fourth row of each subplot).

The down-sampled *ex vivo* data showed a similar level of statistical significance compared to the high resolution data, with a marginal reduction of statistical differences for most of the structures (Figures 4E,F); however, the significance levels of volume changes for a few structures (e.g., the olfactory bulb and the anterior commissure) were altered in the down-sampled data. For the olfactory bulb, the significant difference between treated and untreated rTg4510 groups did not persist after down-sampling, while for the anterior commissure, although no significant difference was detected for both cases, the adjust *p*-value became larger in the down-sampled data, indicating a reduction of statistical power after down-sampling. The fact that even the down-sampled data showed an improved level of significance relative to the *in vivo* analysis indicates that, the improved statistical power in the *ex vivo* data is not solely dominated by the improved resolution (40 um *ex vivo* versus 150 um *in vivo*), but other factors, such as improved CNR.

#### Longitudinal Analysis

When comparing the data from different time points from the *in vivo* data (Figures 4A–C), a pattern of increasing volumetric changes can be observed. Figure 4D shows the w-score and the statistical results of a two-tailed unpaired *t*-test (the adjusted *p*-value shown at the right of the plot) for the longitudinal unnormalized structural volume change rate, calculated from the *in vivo* data, which showed complementary information compared to the single timepoint volume difference (Figures 4A–C). Again, the untreated group is used as the reference group similar to the single-time-point analysis, so the higher values in the treatment subgroup represent better volume preserving effects comparing to the untreated subgroup, therefore reflecting the treatment effect. Significant differences in volume change rate were found in the neocortex, hippocampus, right olfactory bulb, and hypothalamus between the treated and untreated groups. In addition, the longitudinal data showed a higher level of significance of group difference for caudate putamen than the *ex vivo* data (and higher than the *in vivo* data for the right caudate putamen). These differences indicate complementary information over single timepoint *in vivo* and *ex vivo* volumetric analysis.

### Comparison of Multivariate Classification Power

**FIGURE 5:**
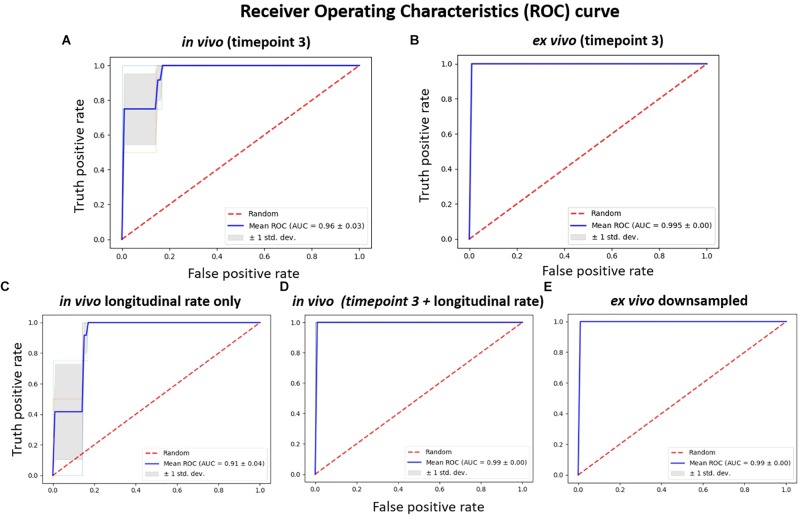
The receiver operating characteristics (ROC) of classification power for: **(A)** *in vivo* volume at timepoint 3 (7.5 months); **(B)** the corresponding *ex vivo* data with original resolution at timepoint 3 (7.5 months); **(C)** longitudinal *in vivo* volume change rate, calculated from the parcellation result of the data acquired at three timepoints (3.5, 5.5, and 7.5 months); **(D)** the *in vivo* feature combining both the single time point volume (at 7.5 months) and longitudinal volume change rate; **(E)** the *ex vivo* volume with volume down-sampled to the same resolution of the *in vivo* data. SVM with linear kernel is used as classifier, and the classification power is represented as the area under the curve (AUC) of the receiver operating characteristic (ROC) for threefold cross-validation.

The results comparing the classification power of the *in vivo* and *ex vivo* data to correctly classify the untreated and treated group of mice using SVM with a linear kernel as the classifier are presented in Figure 5. Threefold cross-validation was performed, and the mean AUC of the ROC are presented as the classification performance, with a larger mean AUC representing better classification power. The *in vivo* data (Figure 5A) showed less classification power when compared with the *ex vivo* data, at either the original resolution (Figure 5B) or down-sampled to the same resolution as *in vivo* data (Figure 5E). In both scenarios, the *ex vivo* classification power showed all-correct prediction with AUC = 1; this can be attributed to the distinctive morphological differences between the two groups that was readily captured *ex vivo*. We noted that the classification power of both the *in vivo* single time-point volumetric analysis (Figure 5A) as well as the *in vivo* longitudinal rate of volumetric change across the three time-points (Figure 5C) demonstrated less classification power relative to the *ex vivo* data; however, the *in vivo* classification power showed marked improvements when these data (both the single time-point and the longitudinal) were combined (Figure 5D). This finding indicates that the two approaches for analysing the *in vivo* data capture complementary information, and the inclusion of both features can improve the classification performance. It is worth mentioning that, although we observed 100% accuracy (mean AUC = 1) for both the combined *in vivo* data (Figure 5D) as well as the two *ex vivo* analyses at original and down-sampled resolution (Figures 5B,E, respectively), this cannot be interpreted as the three set of data showing the same level of classification power.

**FIGURE 6:**
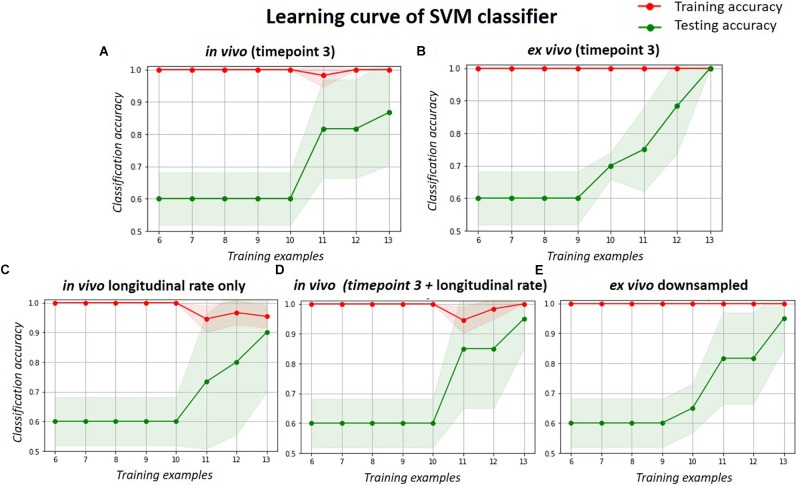
The learning curve of the SVM classifier with respect to different sample size, when classifying the treated and untreated group. Red lines represent the training accuracy, and the green lines represent the test accuracy. The shade region represents the standard deviation. Each subplot shows the classification accuracy for the: **(A)** *in vivo* data; **(B)** *ex vivo* data; **(C)** longitudinal *in vivo* data (in terms of volume change rate); **(D)** combination of the single timepoint cross-sectional and longitudinal *in vivo* data; and **(E)** *ex vivo* data down-sampled to the same resolution of *in vivo* data.

The learning curve (Figure 6) shows the change of classification power to differentiate the doxycycline-treated and untreated rTg4510 groups using the SVM classifier, with respect to different sample size. The testing accuracies of both the *in vivo* and *ex vivo* data remained at 0.60 when the sample sizes were less than 10, and gradually improved with increasing sample sizes. The testing accuracy of *ex vivo* reached 1.00 when the sample size increased to 13 (Figure 6B), while the *in vivo* data with the same sample size only reached a testing accuracy of 0.86 (Figure 6A). For the down-sampled *ex vivo* data, the testing accuracy dropped slightly to 0.95 with a sample size of 13 (Figure 6E). Conversely, the testing accuracy of the longitudinal *in vivo* data increases from 0.60 to 0.90 when the sample size increases from 10 to 13 (Figure 6C). Finally, when the *in vivo* single time-point and longitudinal rate information were combined, the testing accuracy improved to 0.95 when sample size increases to 13; this is comparable to the down-sampled *ex vivo* data.

### Evaluation of Individual Variation in the Longitudinal Scale

**FIGURE 7:**
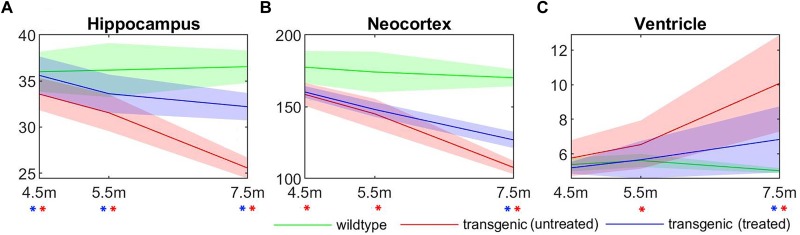
The longitudinal absolute structural volume change of **(A)** hippocampus, **(B)** neocortex, and **(C)** ventricle for all three experimental groups. Green: wild-type mice, red: transgenic mice without treatment, blue: transgenic mice with doxycycline treatment. ^∗^(red): significant volume difference was detected between untreated transgenic and wild-type mice; ^∗^(blue): significant volume difference was detected between treated and untreated transgenic mice. ANOVA with Bonferroni *post hoc* test followed by multiple comparison corrections with FDR = 0.05. Unit of the *y*-axis: mm^3^.

The longitudinal volume change of three structures most affected by AD: hippocampus, neocortex, and ventricle, were plotted in Figure 7, for all three experimental groups: wildtype, rTg4510 mice without treatment, and rTg4510 mice with doxycycline treatment. The longitudinal trend in the result clearly shows the continuous progression of pathologies (wildtype vs. untreated transgenic group), as well as the effect of doxycycline treatment (untreated vs. treated transgenic group) in all three structures. Statistical analysis indicated that the hippocampal/cortical atrophies start to manifest as early as 4.5 months, and the ventricle expansion starts from 5.5 months (as indicated by the significant volume difference between wildtype and untreated transgenic group). In addition, the treatment effect appeared as early as 4.5 months in the hippocampus, and is observable in neocortex and ventricles at 7.5 months (as indicated by the significant volume difference between the treated and untreated transgenic group). These results align with the reported longitudinal disease progression time windows in previously published studies and validate the timepoints selected in this study.

It can be observed from Figure 7 that, compared to the hippocampus and neocortex, the ventricles showed larger individual variation of disease pathology progression, especially in the later timepoints (5.5 and 7.5 months), and exhibit less significant group differences. Therefore, we further analyzed the longitudinal individual variation in the *in vivo* data using LME model. Table 2 shows the result comparing different LEM model performance when evaluating the individual variations in the *in vivo* data. Models performances were evaluated with AIC, and the statistical difference between the corresponding REML estimations. Comparing to the fixed-effect model, the random intercept model performance improves for all three AD-related structures, indicating significant individual variation in the structural volume measurements. The random intercept and slope model showed further performance improvement, demonstrating additional individual variation in the longitudinal volume change rate.

**Table 2:** Comparison of different linear mixed effect (LME) models in terms of AIC and significant difference in REML estimation.

	Akaike information criterion (AIC)			Significance	
**Structures**	**1. Fixed effect**	**2. Random intercept**	**3. Random intercept + slope**	**Model 1 vs. 2**	**Model 2 vs. 3**

Hippocampus	324.78	307.83	301.4	^∗∗^	^∗∗^
Neocortex	512.71	501.54	490.66	^∗∗^	^∗∗^
Ventricle	283.26	272.89	254.27	^∗∗^	^∗∗^


*In each model, the individual variation in the longitudinal scale are modeled as: (1) fixed effect; (2) random intercept of time; (3) random intercept and slope of time. A smaller value of AIC represent better model performance. ^∗∗^*p*-value < 0.001 when comparing two models.*

**FIGURE 8:**
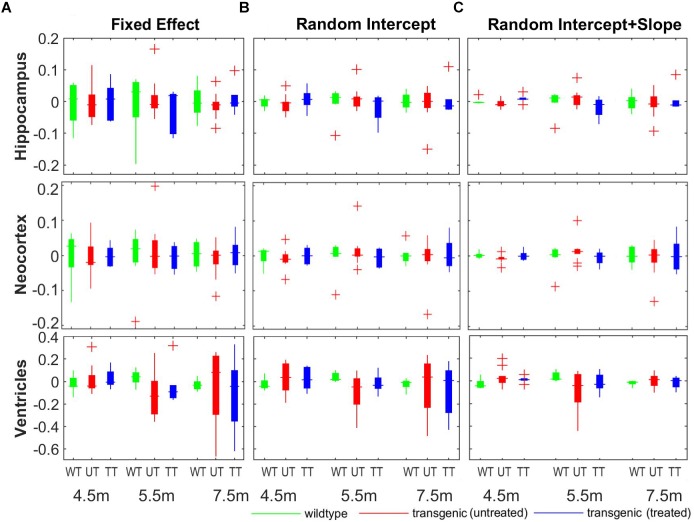
The comparison of the model fitting between the three linear mixed-effect (LME) models. **(A)** Fixed-effect model, in which individual variation was not modeled explicitly, and the longitudinal measurements for each individual subject are modeled as fixed-term; **(B)** random intercept model, in which individual variance on the absolute volume was explicitly modeled by introducing a random-effect term on the intercept; **(C)** random intercept and slope model, in which the individual variance on the longitudinal scale was also modeled by adding an additional random-effect term over the slope of time. The *y*-axis represent the relative residual which is the ratio between the model residual and the actual measured volume (unit: percentage).

Figure 8 shows the comparison of the individual percentage residual of the volumes for all three structures across all three group at different timepoints. The random intercept model (Figure 8B) showed smaller relative residual compared to the fixed-effect model (Figure 8A), while the random intercept and slope model (Figure 8C) reduced the relative residual further, which agree with the model comparison results shown in Table 2. In the fixed-effect model (Figure 8A), the individual variation in the neocortex (middle row) is the smallest among the three structures across all three timepoints, indicating small individual variation in the cortical region. In addition, with the fixed effect model and the random intercept model (Figures 8A,B), the ventricle (bottom row) exhibits larger relative residual compared to hippocampus and neocortex for both the untreated and treated transgenic group (as shown in red and blue box), especially in the later timepoints (5.5 and 7.5 months). This result confirms the larger individual variation in the ventricles in the longitudinal scale. The relative residual is greatly reduced after the individual variance is controlled by including the random effects to the slope of time in the model (Figure 8C, bottom row).

## Discussion

When designing experiments to study diseases using mouse models, one must choose whether to scan animals *in vivo* or *ex vivo*. It is sometimes a controversial choice as each of these imaging paradigms has its own strengths and weaknesses. In this study, we investigated both the progression of longitudinal structural volume changes, as well as the *in vivo* to *ex vivo* volumetric changes due to the preparation of post-mortem tissues. We demonstrated how this choice of paradigm will affect volumetric analysis using automated brain structural parcellation, both in terms of group difference analysis, as well as classification power.

### *In vivo* to *ex vivo* Volumetric Change

Previous studies exploring volumetric changes from *in vivo* to *ex vivo* have shown controversial conclusions. Early histology studies have shown that both perfusion and fixation processes cause tissue shrinkage (Palay et al., 1962; Cragg, 1980). With MRI data, Schulz et al. (2011) investigated the effect of fixation on the volume of the human brain for up to 70 days using image registration, and found unevenly distributed brain shrinkage after initial expansion. Conversely, a study by Kotrotsou et al. (2014) found a linear correlation between *ex vivo* and *in vivo* gray matter volumes, with no significant change during the 6 months’ fixation period. Studies on preclinical imaging data also show inconsistent results. Zhang et al. (2010) used manual segmentation and showed a decrease in *ex vivo* brain volume (4.47% for wild-type mice, and 8% for Huntington’s disease mice). Meanwhile, Ma et al. (2005, 2008) used semi-automatic segmentation propagation and found a 10.6% shrinkage in *ex vivo* brain volumes relative to *in vivo* brain volumes, and reported that some parts of the gray matter shrunk from *in vivo* to *ex vivo* whilst others expanded. However, the *in vivo* and *ex vivo* imaging datasets in these studies were acquired from different mice populations (of the same strain), and the *ex vivo* specimens were scanned after physical skull removal, with brain tissue loss notable from the images. On the other hand, Oguz et al. (2013) performed single-atlas segmentation-propagation on rat brains (male Wistar) but found no significant change between the *in vivo* and *ex vivo* datasets for TBV, and structural volumes. Recent work by Holmes et al. (2017) reported a reduction in the TBV of 10.3% between *in vivo* and *ex vivo* mouse brain MRI and non-uniform morphological change using tensor based morphometry (TBM). Such variance in these different volumetric studies could be attributed to factors such as animal strain difference, pathological model diversity and protocol variation of post-mortem tissue processing. Consequently, these various factors might impact the accuracy and reliability of quantitative measurements extracted from *ex vivo* data.

Our result confirmed the uneven distribution of volumetric changes across different brain structures, as published studies have reported (Ma et al., 2005, 2008; Zhang et al., 2010; Schulz et al., 2011; Holmes et al., 2017). Our study expanded upon these previous findings by quantifying the volume change for each individual structure, in both the gray and white matter. We demonstrated that the post-mortem fixation and perfusion processes introduce different morphological alterations that affects different tissue types: after the *ex vivo* tissue processing, the majority of gray matter structures shrink, while most white matter structures expand. Furthermore, the collapse of almost the entire ventricular space results in a dramatic reduction in CSF volume *ex vivo*. These observed changes in gray and white matter volumes were non-uniformly distributed within each tissue types. Furthermore, our results showed differences in the level of volumetric changes across the three groups of mice: the doxycycline-treated rTg4510s, untreated rTg4510s and wild-type controls. Such variation in volume changes among *ex vivo* tissue types, tissue structures, and between groups, will obviously complicate the interpretation of morphological analysis using techniques such as voxel-based morphometry, which relies on the estimation of proportional volume change between gray matter and white matter. Therefore, although the *ex vivo* volumetric analysis in this study demonstrated superior statistical and classification power for group difference analysis compared to the *in vivo* data acquired at the same time-point, it is, however, difficult to differentiate the proportion of such improvements which represent the true biological effect, and the changes that manifest as a result of the post-mortem tissue processing. For example, the level of *in/ex vivo* volume difference of the superior colliculi is significantly higher in the doxycycline treated group than the untreated group, as shown in Figure 3D. Therefore, the increased *ex vivo* statistical power of the group difference detected within the superior colliculi (as shown in Figures 4C,E) is, in fact, a combination of actual biological morphological differences, and effects originating from the *ex vivo* tissue processing.

Specifically, ventricular expansion is an important neuroimaging biomarker for neurodegenerative diseases such as AD (Nestor et al., 2008; Weiner, 2008). For the *ex vivo* ventricular measurements, the ventricle collapse and the loss of ventricular CSF in the post-mortem brain tissue preservation process. Our study reported a large ventricles volumetric loss from *in vivo* to *ex vivo* which aligns with previous studies: (Ma et al., 2008; Zhang et al., 2010).

The white matter expansion is also interesting, which indicates potential microstructural-level volume expansion of the white matter tract. Compared to the GM, the WM contains significantly less water (∼70% vs. ∼85%) and more lipid (16–22/100 g vs. 5–6/100 g) (Davison and Wajda, 1962). The post-mortem brain tissue fixation changes various MR indexes significantly, such as T1, the magnetization transfer ratio (MTR), and the macromolecular protons fraction, which also differs between GM and WM (Schmierer et al., 2008, 2010). Such compositional and signal difference will obviously affect the volume change to different tissues types. Von Halbach und Bohlen et al. (2014) proposed an alternative method to conduct post-mortem *ex vivo* imaging directly after the animal has been sacrificed, to prevent the potential volume changes associated with the preparation of fixed tissues. However, such *ex vivo* imaging procedure is inevitably contaminated by the fast post-mortem tissue degradation (Sun et al., 2015). This becomes even more significant given the long scanning time of the *ex vivo* imaging which easily adds up to several hours. In addition, tissue samples frequently undergo histological evaluation after *ex vivo* imaging to corroborate structural changes with alterations occurring at the cellular level. The advanced brain tissue decomposition after long scans will also affect the quality of the histology evaluation (Von Halbach und Bohlen et al., 2014).

It is worth noting that, the measurements for the *ex vivo* images depend highly on the post-mortem tissue processing protocol in MR microscopy, In the case of this study, the in-skull brain tissue was soaked in contrast-enhanced agent for 9 weeks before *ex vivo* imaging, which would theoretically aggravate the tissue dehydration. The protocol diversity among various *ex vivo* studies should account for a large portion of the difference in the corresponding results. Therefore, the *ex vivo* brain structure volume change reported in the current paper should be regarded as specific to the active staining tissue processing protocol used in this study. On the other hand, such variation in the results of different *ex vivo* studies emphasize the importance and advantage of protocol consistency for *in vivo* measurements.

### Comparison Between *in vivo* and *ex vivo* Morphological Analysis

Lerch et al. (2012) have compared the theoretical statistical power between *in vivo* and *ex vivo* imaging, using a pre-determined variance value with simulated deformation on the hippocampus. Their result showed that *ex vivo* imaging provides better precision and should be preferred if the volume is normalized to TBV, as the normalization process regress out the effect of gross brain volume difference between individual animals; while *in vivo* measurements give better results on absolute volume measurements and can provide more accuracy in longitudinal studies than cross-sectional *ex vivo* measurements. In a recent study, Holmes et al. (2017) have conducted power analysis to determine the required sample size in order to detect a specific amount of local morphological variation either *in vivo* or *ex vivo*. Careful power analysis is important to determine the appropriate sample size given effect size. Comparing with voxel-wise statistical analysis such as TBM (Holmes et al., 2017), the required sample size to detect volume difference is smaller for structural-based analysis, as the effect toward all the voxels in each structure are grouped together if the intra-structural volume change is homogenous enough. Our findings extended these theoretical analyses with application to longitudinal *in vivo* analysis on each individual structural volume. Although the longitudinal analysis based on structure volume change rate by itself is less powerful to statistically compare and classify different groups, it indeed showed complementary information over the single-time-point volume information. We showed that by combining the both the longitudinal and cross-sectional *in vivo* volumetric information, there is an improvement of the classification power.

In this study, we modeled the longitudinal volume change as a liner effect for each all structures. However, the change of ratio might be in fact not linear, and the time that volume change occurs can be different for different structures (either due to the nature of the pathological process, or the treatment start to show effect). Therefore, a model alternative to the linear regression would potentially represent the actual volume change better and further improved the *in vivo* analysis results. Furthermore, by integrating the volume information with other *in vivo* assessment to form multimodality analysis (such as CEST and CBF (Wells et al., 2015; Holmes et al., 2017) could potentially further improve the statistical and classification power for the *in vivo* data.

Specifically, it is interesting that both the *in vivo* and *ex vivo* data of the transgenic mouse at 7.5 months showed cerebellar shrinkage after doxycycline treatment. The cerebellum is traditionally considered unaffected in AD, although recent studies have shown increase evidence that it is also affected during the AD disease progression (Larner, 1997; Jacobs et al., 2018), which is also the case for rTg4510 (Xie et al., 2010). However, further investigations are required to draw connection between potential neuroprotective or neurotoxicity effect of doxycycline to the cerebellum, such as the cerebellar plasticity (Ito, 2012), to help us understand the observation reported in this study.

In this study, we used the w-score (Eq. 3, Figure 4) to visualize the group difference rather than the raw volume. The advantage of using w-score is that the volume measurements of each individual structure are transformed to the reference group mean and normalized by the reference group standard deviation. This process standardizes the group difference for all the features to the same scale, effectively improve the feature-based classification (Fortin et al., 2017; Rozycki et al., 2017).

In experiments with biological tissues or subjects, the variations of the individual measurements are often observed. The presence of outliers may affect the power of statistical analysis, especially in cases where the sample number is relatively small This is a common issue that animal studies usually suffer from, especially when the effect size of the group difference is small. In this study, we presented a data visualization method that is capable of pooling the entire dataset within a panorama figure showing multiple measurements for each individual (as shown in Figures 3, 4). Moreover, presenting the w-score of the raw measurement ensures meaningful visualization of the individual variation while preserving the statistical analysis results, since all the data are shifted and scaled by the same number (i.e., the mean and standard deviation of the reference group). Such data visualization technique is an intuitive way to demonstrate internal data inhomogeneity on very large databases (Ma et al., 2018), and in this study, showed individual variations in small dataset as well. In addition, comparison of different LME models demonstrated region-specific, group-dependent, and time-variate individual variations in the longitudinal *in vivo* measurement of structure volume.

When classifying the treated and untreated group, the SVM showed satisfactory results even with the relatively small sample size (Figures 5, 6), thanks to the relatively large effect size between the two groups. Never the less, the classification power analysis result clearly demonstrated the improvement in testing accuracy after combining cross-sectional and longitudinal *in vivo* data when comparing with *ex vivo* data, although larger sample size is required to reduce the testing error when the effect size between the groups is small (Figueroa et al., 2012; Beleites et al., 2013). Techniques such as bootstrap aggregating (a.k.a bagging) can be used, along with increasing the number of data, to reduce the variance in the training, improve the classification accuracy, and avoid overfitting (Dietterich, 2000).

In the field of preclinical imaging research, we anticipate that the widely regarded ‘gold standard’ for investigating mouse models at the macroscopic level to shift from histology to *ex vivo* imaging, and later to *in vivo* imaging. Such a shift in the imaging paradigm will not only enable the longitudinal assessment of neuroanatomical changes but will also help reduce the number of animals dedicated to preclinical studies (Gunn et al., 2012; Home Office, 2014).

### Limitations of the Current Study

In the current study, we used TBV as a normalization term. However, the TBV itself is a dependent variable toward the treatment effect (Holmes et al., 2017). A better alternative to normalized the data should be estimating the total intracranial volume (TIV) employing tissue classification techniques, which use expectation maximization to estimate the tissue probability for each voxel, including gray matter, white matter, CSF, and non-brain tissues, and estimate the TIV as the summation of all types of brain tissues (Lemieux et al., 2003; Acer et al., 2007; Ridgway et al., 2011). However, a tissue probability map is necessary as prior information to initialize the expectation-maximization procedure. One of the current limitations in mouse brain MRI studies is the lack of such accurate tissue probability map. A tissue classification framework with accurate tissue probability maps (Sawiak et al., 2013; Powell et al., 2016; Hikishima et al., 2017) would be beneficial for future preclinical studies.

In the section of classification power comparison, no feature selection and hyperparameter tuning were performed, and the selected models and hyperparameters do not necessarily reflect the optimal choice or value for the group classification for the current dataset. However, model optimization is not the focus of this study, and the main purpose of this section of analysis is to compare the classification performance using the same model and parameter when applied from the dataset collected from the same sample but with different measurement (*in vivo* versus *ex vivo*).

Furthermore, in the current study, the *in vivo* and *ex vivo* images were acquired using different imaging protocols with different scanning sequences and coils, for comparing the best quality of each. Consequently, the measured *in/ex vivo* volume difference is a combination of the biological/pathological change and the measurement difference due to different image quality (e.g., CNR) between the *in vivo* and *ex vivo* images. In an ideal experiment setup, the same scanning protocol (i.e., the same coil and scanning sequence) would be used to acquire both *in vivo* and *ex vivo* images in order to have a bias-free comparison to assess the volume change accurately. This will on one hand effectively eliminate some confounding factors from the images, but on the other hand, losing its representation of the best image quality acquired in real practice. One effort to alleviate such bias in this study is to down-sample the *ex vivo* data to the same resolution of the *in vivo* data, which reduces the bias that comes directly from the resolution difference. On the other hand, even with the similar resolution, the higher GM/WM CNR in the *ex vivo* data, as shown in Table 1, helps to improve the automatic structural parcellation accuracy, and demonstrated higher statistical and classification power.

In this study, only female mice are used to control the effect of sex toward the variation of the data. However, sex-specific differences have been reported in AD (Mielke et al., 2014; Mazure and Swendsen, 2016; Laws et al., 2018), such as faster cognitive decline and pathological progression in female than male (Ferretti et al., 2018). Specifically, the rTg4510 mice model also showed significantly higher levels of Tau-induced pathology in female mice at 5.5 months (Yue et al., 2011). Therefore, the result and conclusion presented in this study can only be referred to females, and data from both sexes are required to draw more generous conclusions about the disease specification and potential treatment effect for precision medicine.

Finally, the variation comes from scanning gradient coil difference can be alleviated through careful gradient calibration. Gradient calibration is crucial for MRI to eliminate any time-dependent gradient shift to ensure the acquired image represented the tissue volumes accurately. This is especially important for longitudinal studies across a long period of time, as well as the comparison between images acquired with different gradient coil, as in the case of our study. However, unlike clinical systems, the frequency of gradient calibration for the preclinical system is sometimes insufficient. By employing the gradient calibration protocol we developed previously and employed in this study (O’Callaghan et al., 2014), we detected that the 72 mm birdcage radiofrequency (RF) coil we used for *in vivo* scan comes with around 0.1% gradient shift per month, which will cause significant system bias for both longitudinal analysis using *in vivo* data, as well as analysis comparing *in vivo* and *ex vivo* data acquired from different gradient coil. The effect of such longitudinal imaging gradients shift has been alleviated through proper gradient calibration, and the associated biases have been removed prior to any longitudinal and cross-sectional analysis.

## Conclusion

In conclusion, in this paper, we presented our study to compare the volumetric analysis for longitudinal *in vivo* imaging and cross-sectional *ex vivo* imaging using automated mouse brain MRI structural parcellation. We showed non-uniformly distributed structural volume changes from *in vivo* to *ex vivo* measurements across different tissue types. We also demonstrated the effect of mouse strains and drug treatment toward the *in vivo* to *ex vivo* volume change. Our result demonstrated higher statistical and classification power using the *ex vivo* structure volume compared to the *in vivo* counterpart, although the volume differences in the *ex vivo* data represent a combination of both the biological/physiological effect as well as the effect due to post-mortem tissue processing. On the other hand, the *in vivo* measurements identified ventricular shrinkage, while *ex vivo* measurements were not sensitive to these changes due to the ventricular collapse during the preparation of the post-mortem tissues. In addition, we showed that the longitudinal *in vivo* imaging provided complementary information other than single-timepoint measurement. Incorporating the information obtained from the longitudinal data as additional features significantly improves the classification power.

## Ethics Statement

All studies were carried out in accordance with the recommendations of United Kingdom’s Animals (Scientific Procedures) Act of 1986 and approved by the UCL internal ethics committee.

## Author Contributions

ML, SO, MM, and MC contributed to the conception and design of the study, provide infrastructure, and provide overall supervision. RJ, MO, and EC designed and advised on the animal experiment design and treatment experiment design. HH performed the data acquisition experiments, optimize the protocol, and acquired the data. OI manages the experimental setup as well as the treatment procedure. DM, NP, and JO performed the first phase of data processing and analysis. DM, MB, and KP contributed and performed the second batch of the data analysis and visualization. DM wrote the first draft of the manuscript. HH, IH, and NP refined the experimental setup and data analysis protocol. All authors contributed to the manuscript revision, read and approved the submitted version.

## Conflict of Interest Statement

The authors declare that the research was conducted in the absence of any commercial or financial relationships that could be construed as a potential conflict of interest.
